# The Question of Repetition

**Published:** 1893-07

**Authors:** Howard Crutcher

**Affiliations:** Chicago


### THE QUESTION OF REPETITION.

HOWARD CRUTCHER, M. D., CHICAGO.

A few days ago while speeding along on a fast railway train a sleepy-headed person yawned, rubbed his eyes, and, inquiring of himself how far a certain station was ahead, took out his watch, remarking as he did so: "I will see where we are."

A traveling companion inquired the cause of my overflowing merriment.

"What a magnificent mongrel that fellow would make," I replied. "Instead of looking out the window at the country, with which he may be familiar, or looking at the mile-posts along the right of way, he looks at his watch to find out where he is! The man who gives medicine every hour or every half-hour or every two hours or every night and morning ought not to do much laughing at that fellow. One man neglects the mile-posts and the other neglects the patient. They are both in the same boat."

